# Increased community-acquired upper urinary tract infections caused by extended-spectrum beta-lactamase-producing *Escherichia coli* in children and the efficacy of flomoxef and cefmetazole

**DOI:** 10.1007/s10157-019-01775-w

**Published:** 2019-08-19

**Authors:** Akiyoshi Horie, Akiyoshi Nariai, Fumihide Katou, Yasuhiro Abe, Yuya Saito, Daisuke Koike, Tomohiro Hirade, Tomoko Ito, Miho Wakuri, Aiko Fukuma

**Affiliations:** 1 Division of Pediatrics, Shimane Prefectural Central Hospital, 4-1-1 Himebara, Izumo, Shimane 693-8555 Japan; 2 Division of Neonatology, Shimane Prefectural Central Hospital, Izumo, Japan; 3 Division of Bacteriology, Shimane Prefectural Central Hospital, Izumo, Japan; 4 Division of Bacteriology, LSI Medience Corporation, Tokyo, Japan; 5 Division of Bacteriology, Shimane Prefectural Institute of Public Health and Environmental Science, Matsue, Japan

**Keywords:** Upper urinary tract infection, Extended-spectrum beta-lactamase-producing *escherichia coli*, Flomoxef, Cefmetazole

## Abstract

**Background:**

Urinary tract infections caused by extended-spectrum beta-lactamase-producing bacteria are increasing worldwide. At our hospital, the number of pediatric patients hospitalized because of an upper urinary tract infection has dramatically increased since 2016. In total, 60.5% of urinary tract infections are caused by extended-spectrum beta-lactamase-producing *Escherichia coli*. Such a high prevalence of extended-spectrum beta-lactamase-producing *E. coli* has not been detected previously in Japan. Therefore, we evaluated the clinical and bacteriologic characteristics and efficacy of antibiotics against upper urinary tract infections caused by *E. coli* in children.

**Methods:**

This retrospective study surveyed 152 patients who were hospitalized in the pediatric department of Shimane Prefectural Central Hospital because of upper urinary tract infections caused by *E. coli*. Medical records were reviewed to examine patient characteristics. O antigens, antibiotic susceptibility, gene typing, and pulse-field gel electrophoresis were studied at the Shimane Prefectural Institute of Public Health and Environmental Science.

**Results:**

Urine sample analyses showed extended-spectrum beta-lactamase types such as CTX-M-9 and plural virulence genes. We changed the primary antibiotic treatment to flomoxef or cefmetazole to treat upper urinary tract infections caused by Gram-negative bacilli. After changing treatment, the time to fever alleviation was significantly shortened.

**Conclusion:**

Extended-spectrum beta-lactamase-producing *E. coli* should be suspected in community-acquired upper urinary tract infections. Therefore, when treating patients, it is necessary to focus on antibiotic susceptibility and the prevalence of extended-spectrum beta-lactamase-producing bacteria found in each area. Flomoxef and cefmetazole are useful primary treatments for upper urinary tract infections caused by extended-spectrum beta-lactamase-producing *E. coli*.

## Introduction

The prevalence of extended-spectrum beta-lactamase (ESBL)-producing bacilli has increased dramatically worldwide [1, 2], and the CTX-M-type of ESBLs is the most frequent type [1, 3, 4]. ESBL-producing bacilli often exhibit multi-drug resistance, and related genes are encoded by plasmids that are transferred from species to species.

Previously, ESBL-producing bacteria have caused nosocomial infections, but now they also cause community-acquired infections; moreover, reports of urinary tract infections caused by ESBL-producing bacteria in children have increased [5]. Generally, upper urinary tract infections (UUTI) are primarily caused by enterobacteria that colonize in the rectum, which indicates a fecal–perineal–urethral route of infection [6].

In 2016, in Izumo City, Japan, the number of pediatric patients hospitalized because of UUTI caused by ESBL-producing *E. coli* dramatically increased. *E. coli* strains isolated from the urinary tract are known as uropathogenic *E. coli* (UPEC). Virulence genes have been involved in UPEC infections, fimbria, adhesions, toxins, lipopolysaccharides, and capsules, which facilitate the colonization of bacteria in the urinary tract and invasion in host cells [7]. Outbreaks of UUTI have been suspected to be caused by UPEC acquired by an ESBL-producing gene. Therefore, we aimed to determine the clinical and bacteriological characteristics of ESBL-producing *E. coli* that cause community-acquired UUTI in children and how to cope with the situation.

## Patients and methods

### Patients

Data used for this study were retrospectively collected from medical records in an electronic database at Shimane Prefectural Central Hospital. We collected medical records of patients hospitalized with UUTI caused by bacteria and who were admitted to the pediatric department between January 1, 2011, and December 31, 2018. The medical records were reviewed for age at hospitalization, sex, history of hospitalization, degree of vesicoureteral reflux (VUR) detected by voiding cystogram, white blood cell (WBC) count, C-reactive protein (CRP) levels, and procalcitonin (PCT) levels at the time of hospitalization. The bacterial profile was determined using urine culture results, stool culture results, and antibiotic treatments. UUTI were diagnosed based on the finding of more than 10^4^ CFU/mL bacteria in urine caught by a catheter and clinical symptoms. The primary outcome was defined as time (hours) after antibiological treatment was started until fever was alleviated (37.5 °C). Infection relapse was defined as the presence of bacteria in urine and associated clinical symptoms within 1 month after the completion of the primary treatment. Exclusion criteria were treatment with an immunosuppressive drug, history of UUTI, and major congenital anomalies. Additionally, we excluded patients with suspected bacteremia and sepsis who were treated with carbapenems at onset.

Microbiological identification of *E. coli* was performed using the VITEK 2 GN identification card. Screening of ESBL production was performed using an assumed ceftriaxone (CTRX)-resistant strain with ESBL-producing bacteria and an identification test disk (AmpC/ESBL differentiation disk; Kanto Chemical Co., Inc.); differentiation of ESBL-producing bacteria was based on the criteria of the Clinical and Laboratory Standards Institute (CLSI). Antibiotic susceptibility was evaluated according to the definitive method of the CLSI. Drug sensitivity was examined by performing agar plate diffusion based on the CLSI method. The O antigen of *E. coli* was determined using an enteropathogenic *E. coli* immune serum (Seiken; Denka Seiken Co., Ltd.).

Shimane Prefectural Institute of Public Health and Environmental Science Center analyzed the ESBL-producing *E. coli* found in the urine and blood cultures. Analyses of ESBL types, O antigens, drug resistance, virulence genes, and pulse-field gel electrophoresis (PFGE) were performed. To determine the ESBL genotypes, a polymerase chain reaction was performed using primers specific for TEM, SHV, the CTX-M-1 group, the CTX-M-2 group, and the CTX-M-9 group, as reported previously [8]. Polymerase chain reaction was used to detect virulence genes, including uropathogenic-specific protein (*usp*), S fimbriae (*sfaD/C*), the outer membrane protein of P fimbriae (*pspC*), aerobactin (*iucD*), type 1 fimbriae (*fimH*), group II capsule (*kpsMT II*), cytotoxic necrotizing factor type 1 (*cnf1*), and α-hemolysin (*hlyA*) [7]. A genotypic analysis based on PFGE was performed using the restriction enzyme Xbal, as reported previously [9].

All patients included in this study were hospitalized for UUTI caused by *E. coli*. Based on *E. coli* detection in urine cultures and antibiotics used for treatment at hospitalization, these patients were divided into five groups: (1) ESBL-nonproducing *E. coli* CTX group [UUTI caused by ESBL-nonproducing *E. coli* and treated with CTX sodium (100 mg/kg/day) or CTRX disodium (60 mg/kg/day)]; (2) ESBL-producing *E. coli* CTX group (treatment with CTX sodium or CTRX disodium); (3) ESBL-nonproducing *E. coli* FMOX or CMZ group [treatment with flomoxef (80 mg/kg/day) or cefmetazole (100 mg/kg/day)]; (4) ESBL-producing *E. coli* FMOX group (treatment with flomoxef); and (5) ESBL-producing *E. coli* CMZ group (treatment with cefmetazole). All antimicrobial drugs were administered intravenously at their usual doses.

### Statistical analysis

The Kruskal–Wallis *H* test and Mann–Whitney *U* test were used to compare age, WBC, CRP, PCT, and time (hours) after the start of antibiotic treatment until fever alleviation. Values were expressed as mean ± standard deviation (SD). The Chi-squared test was used to determine the correlation between sex, history of hospitalization, VUR, blood cultures, and relapse. For all analyses, *p* < 0.05 was considered statistically significant.

## Results

**Fig. 1:**
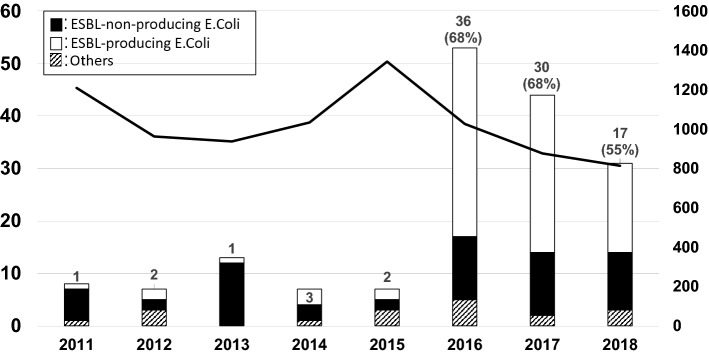
Incidence and cause of upper urinary tract infections. Hospitalized patients with an upper urinary tract infection caused by ESBL-producing *E. coli* (white bars and left *Y*-axes) and the total number of patients hospitalized in our department (line chart and right *Y*-axes) are shown. *ESBL* extended-spectrum beta-lactamase

One hundred seventy UUTI patients who were admitted to the department of pediatrics at our hospital and who met the inclusion criteria were enrolled in the study. UUTI progress in children is shown in Fig. 1. UUTI cases increased dramatically in 2016, and the most frequent cause was *E. coli* (152 cases). However, 60.5% of cases were caused by ESBL-producing *E. coli*. Among 152 UUTI cases caused by *E. coli*, 60 were caused by ESBL-nonproducing *E. coli* and 92 were caused by ESBL-producing *E. coli*. Patient demographics and examination data at the time of hospitalization are summarized in Table 1. There were no significant differences in sex, age at the time of hospitalization, or the number of patients who were born at our hospital. Patients with a history of hospitalization were more likely to have infections caused by ESBL-producing *E. coli*. Examination data at the time of hospitalization indicated no significant differences in WBC, CRP, PCT, or relapse number. Although not significant, bacteremia cases were increased in the ESBL-producing *E. coli* group.

**Table 1:** Demographics, clinical characteristics, and examination data of pediatric patients with UUTI caused by *E. coli* at the time of hospitalization

	ESBL-producing *E. coli*	ESBL nonproducing *E. coli*	*P* value
*n*	93	60	
Male	60	35	0.39
Age (month)	0–159 (median 4)	0–120 (median 4)	0.39
Born at our hospital	36	21	0.73
VUR (greater than grade 2)	9/51	16/41	0.039
History of hospitalization	18 (19.6%)	3 (5%)	0.021
WBC (/µl)	17,923 ± 6379	17,609 ± 6562	0.77
CRP (mg/dl)	8.90 ± 5.02	6.70 ± 5.86	0.15
PCT (mg/dl)	5.62 ± 9.60	3.85 ± 8.38	0.12
Case of bacteremia	9/84 (10.7%)	3/52 (5.8%)	0.49
Case of relapse	10	7	0.91

*WBC* white blood cell, *CRP* C-reactive protein, *PCT* procalcitonin, *VUR* vesicoureteral reflux

Molecular and genetic examinations of these patients were performed. An analysis was performed for nine ESBL-producing *E. coli* samples randomly isolated from pediatric UUTI patients in 2017. Among the nine sample, eight were urine cultures and one was a blood culture. Results of the detected O antigens, types of ESBL genes, antibiotic susceptibility, and virulence genes are shown in Table 2. Among eight of the nine samples, O6 antigen and antibiotics had the same susceptibility to ABPC, CTX, and ST resistance and a tendency for multi-drug resistance. The ESBL genotype of all samples was CTX-M-9, and eight of nine samples with the same O6 antigen exhibited *bla*_CTX-M-27_. Plural virulence genes that were uropathogenic were detected. Eight of nine samples with an O6 antigen, *bla*_CTX-M-27_, and the same susceptibility to drugs exhibited the same virulence genes (*fimH*, *pspC*, *usp*, *hlyA*, and *iucD*). Furthermore, a PFGE analysis showed similarity of more than 90% for these samples. ESBL-producing *E. coli* that caused UUTI in children had the same type of ESBL-producing gene and pathogenicity as UPEC.

**Table 2:** O antigen, CTX-M group typing, virulence gene, and antimicrobial resistance profiles of ESBL-producing *E. coli* samples

Sample number	O antigen	CTX-M-type ESBL	*fimH*	*papC*	*sfaC/D*	*hlyA*	*cnf-1*	Virulence genes			Antibiotic resistance
								*kpsMT II*	*iucD*	*usp*	
1	6	CTX-M-27	** + **	** + **	**−**	** + **	**−**	**−**	** + **	** + **	ABPC,CTX,ST
2	6	CTX-M-27	** + **	** + **	**−**	** + **	**−**	**−**	** + **	** + **	ABPC,CTX,ST
3	6	CTX-M-27	** + **	** + **	**−**	** + **	**−**	**−**	** + **	** + **	ABPC,CTX,ST
4	6	CTX-M-27	** + **	** + **	**−**	** + **	**−**	**−**	** + **	** + **	ABPC,CTX,,ST
5	UT	CTX-M-14 (TEM-1)	** + **	** + **	**−**	**−**	**−**	** + **	**−**	** + **	ABPC,CTX
6	6	CTX-M-27	** + **	** + **	**−**	** + **	**−**	**−**	** + **	** + **	ABPC,CTX,ST
7	6	CTX-M-27	** + **	** + **	**−**	** + **	**−**	**−**	** + **	** + **	ABPC,CTX,ST
8	6	CTX-M-27	** + **	** + **	**−**	** + **	**−**	**−**	** + **	** + **	ABPC,CTX,ST
9	6	CTX-M-27	** + **	** + **	**−**	** + **	**−**	**−**	** + **	** + **	ABPC,CTX,ST

Type 1 fimbriae (*fimH*), outer membrane protein of P. fimbriae (*pspC*), S. fimbriae (*sfaD/C*), α-hemolysin (*hlyA*), cytotoxic necrotizing factor type 1 (*cnf1*), group II capsule (*kpsMT II*), aerobactin (*iucD*), and uropathogenic-specific protein (*usp*)

Ampicillin (ABPC), cefotaxime (CTX), sulfamethoxazole-trimethoprim (ST)

**Fig. 2:**
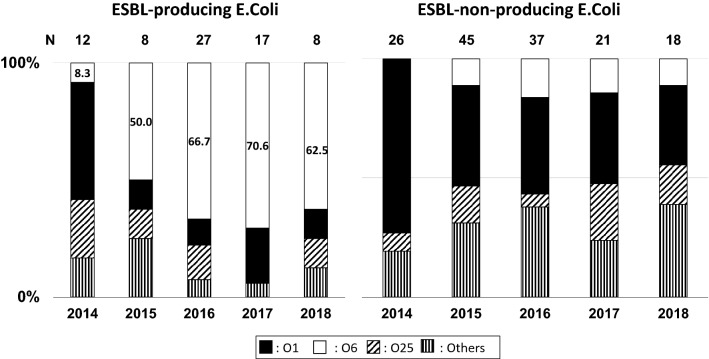
Prevalence of the O antigen among extended-spectrum beta-lactamase-producing *E. coli* detected in a stool sample. The rate of O antigens with extended-spectrum beta-lactamase-producing *E. coli* is shown

Until 2014, O antigens of ESBL-producing *E. coli* were detected in stool samples (Fig. 2); O1, O6, and O25 antigens of *E. coli* were detected, and these antigens were considered uropathogenic. The rate of ESBL-producing *E. coli* with the O6 antigen significantly increased. The antibiotic susceptibility of *E. coli* with each O antigen is shown in Table 3, and that of ESBL-producing *E. coli* detected in pediatric urine samples is shown in Table 4. ESBL-producing *E. coli* detected in urine samples was similar to ESBL-producing *E. coli* in stool samples that had the O6 antigen.

**Table 3:** Antimicrobial resistance profiles of ESBL-producing *E. coli* in each O antigen detected in pediatric stool samples (2016–2018)

O antigen	ESBL-producing *E.coli*			ESBL nonproducing *E.coli*		
	O1	O6	O25	O1	O6	O25
*n*	27	17	8	37	21	18
Antibiotic susceptibility (%)						
ABPC	0	0	0	82.1	93.8	63.2
PIPC	0	0	0	83.6	93.8	68.4
TAZ/PIPC	100	100	100	100	100	100
SBT/ABPC	12.5	100	80	85.1	93.8	68.4
CEZ	0	0	0	94	100	94.7
CAZ	0	0	0	98.5	100	100
CTRX	0	0	0	95.5	100	100
CFPM	0	0	0	98.5	100	100
CMZ	100	100	100	100	100	100
IPM	100	100	100	100	100	100
MEPM	100	100	100	100	100	100
AZT	0	0	0	98.5	100	100
TOB	100	100	100	98.5	100	94.7
AMK	100	100	100	100	100	100
CPFX	87.5	100	0	100	100	100
LVFX	87.5	100	0	98.5	100	63.2
ST	75	5.7	40	98.5	100	63.2
SBT/CPZ	12.5	97.1	80	100	81.3	84.2
FOM	100	100	80	97	100	100

Ampicillin (ABPC), piperacillin (PIPC), tazobactam/piperacillin (TAZ/PIPC), sulbactam/ampicillin (SBT/ABPC), cefazolin (CEZ), ceftazidime (CAZ), ceftriaxone (CTRX), cefepime (CFPN), cefmetazole (CMZ), imipenem (IPM), meropenem (MEPM), aztreonam (AZT), tobramycin (TOB), amikacin (AMK), ciprofloxacin (CPFX), levofloxacin (LVFX), sulfamethoxazole-trimethoprim (ST), sulbactam/cefoperazone (SBT/CPZ), fosfomycin (FOM) are shown

**Table 4:** Antimicrobial resistance profiles of ESBL-producing *E. coli* detected in pediatric urine samples each year

Year	ESBL-producing *E. coli*				
	2014	2015	2016	2017	2018
*n*	3	2	36	30	17
Antibiotic susceptibility (%)					
ABPC	0	0	0	0	0
PIPC	0	0	0	0	0
TAZ/PIPC	75	100	100	100	100
SBT/ABPC	25	60	88.6	93.3	90
CEZ	0	0	0	0	0
CAZ	0	0	0	0	0
CTRX	0	0	0	0	0
CFPM	0	0	0	0	0
CMZ	91.7	100	100	100	100
IPM	100	100	100	100	100
MEPM	100	100	100	100	100
AZT	0	0	0	0	0
TOB	75	80	97.1	100	100
AMK	100	100	100	100	100
CPFX	50	100	97.1	96.7	95
LVFX	50	100	97.1	96.7	95
ST	58.3	0	8.6	20	5
SBT/CPZ	25	60	88.6	93.3	90

Ampicillin (ABPC), piperacillin (PIPC), tazobactam/piperacillin (TAZ/PIPC), sulbactam/ampicillin (SBT/ABPC), cefazolin (CEZ), ceftazidime (CAZ), ceftriaxone (CTRX), cefepime (CFPN), cefmetazole (CMZ), imipenem (IPM), meropenem (MEPM), aztreonam (AZT), tobramycin (TOB), amikacin (AMK), ciprofloxacin (CPFX), levofloxacin (LVFX), sulfamethoxazole-trimethoprim (ST), sulbactam/cefoperazone (SBT/CPZ) are shown

We changed the primary antibiotic treatment to cephamycins such as FMOX or CMZ to treat UUTI caused by Gram-negative bacilli. Of the 60 patients hospitalized with UUTI caused by ESBL-nonproducing *E. coli*, 21 were treated with CTX sodium or CTRX disodium (ESBL-nonproducing *E. coli* CTX group), 33 with cephamycins (26, FMOX; 7, CMZ; ESBL-nonproducing *E. coli* FMOX or CMZ group), and 6 with other antibiotics. Of the 92 patients who were hospitalized with UUTI caused by ESBL-producing *E. coli*, 16 were treated with cefotaxime sodium or CTRX disodium at onset (ESBL-producing *E. coli* CTX group), 53 were treated with FMOX (ESBL-producing *E. coli* FMOX group), 9 were treated with CMZ (ESBL-producing *E. coli* CMZ group), and 14 were treated with other antibiotics. Patient demographics are summarized in Table 5.

**Table 5:** Examination data of patients treated with each antibiotic

	ESBL-nonproducing EC CTX	ESBL-nonproducing EC FMOX or CMZ	ESBL-producing EC CTX	ESBL-producing EC FMOX	ESBL-producing EC CMZ
*n*	21	33	16	53	9
Male	12	19	10	32	6
Age (month)	0–108 (median 3)	0–95 (median 4)	1–132 (median 4)	1–159 (median 5)	1–15 (median 3)
WBC	17,750 ± 6252	18,328 ± 6214	17,786 ± 6348	18,506 ± 6789	15,435 ± 6307
CRP	5.74 ± 4.91	5.98 ± 4.74	7.04 ± 4.24	9.84 ± 5.14	5.65 ± 3.78
Change the antibiotics case	0	0	4	1	1

ESBL-nonproducing EC-CTX upper urinary tract infection (UUTI) caused by extended-spectrum beta-lactamase not producing *E. coli* treated with cefotaxime sodium (CTX) or ceftriaxone disodium (CTRX); ESBL-nonproducing EC-FMOX or CMZ upper urinary tract infection (UUTI) caused by extended-spectrum beta-lactamase not producing *E. coli* treated with flomoxef (FMOX) or cefmetazole (CMZ); ESBL-producing EC-CTX UUTI caused by ESBL-producing *E. coli* treated with CTX or CTRX; ESBL-producing EC-FMOX UUTI caused by ESBL-producing *E. coli* treated with FMOX; and ESBL-producing EC-CMZ UUTI caused by ESBL-producing *E. coli* treated with CMZ are shown

*WBC* white blood cell, *CRP* C-reactive protein

**Fig. 3:**
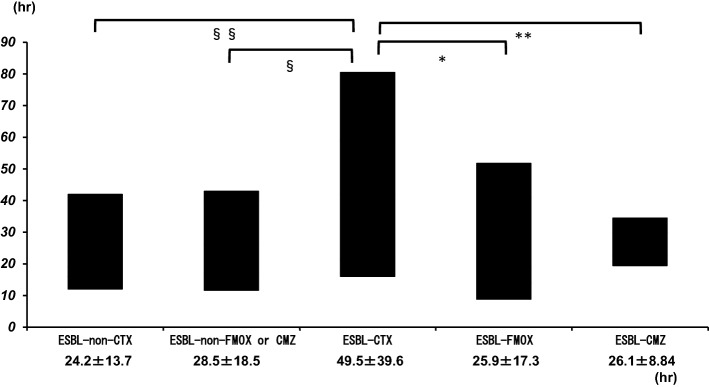
Time until fever was alleviated after treatment with each type of antibiotic. The following treatments are shown: ESBL-non-CTX, upper urinary tract infection (UUTI) caused by extended-spectrum beta-lactamase (ESBL)-nonproducing *E. coli* and treated with cefotaxime sodium (CTX) or ceftriaxone disodium (CTRX); ESBL-CTX, UUTI caused by ESBL-producing *E. coli* and treated with CTX or CTRX; ESBL-non-FMOX or CMZ, UUTI caused by ESBL-nonproducing *E. coli* and treated with flomoxef (FMOX) or cefmetazole (CMZ); ESBL-FMOX or CMZ, UUTI caused by ESBL-producing *E. coli* and treated with FMOX or CMZ. *, **, §, §§, *p* < 0.05

In the ESBL-producing *E. coli* CTX group, more patients required an antibiotic change to a second treatment with carbapenems than in the ESBL-nonproducing *E. coli* CTX, ESBL-nonproducing *E. coli* FMOX or CMZ, and ESBL-producing *E. coli* FMOX groups (*p* < 0.05). However, no patients showed poor outcomes during this study period. We evaluated the time (hours) until fever was alleviated after beginning each antibiotic treatment (Fig. 3). There were significant differences according to the Kruskal–Wallis *H* test for UUTI caused by ESBL-producing *E. coli*. Fever was alleviated at 49.5 ± 39.6 h after treatment with cefotaxime sodium or CTRX disodium (ESBL-producing *E. coli* CTX group), which was significantly longer than that for fever with UUTI caused by ESBL-nonproducing *E. coli* [ESBL-nonproducing *E. coli* CTX group: 24.2 ± 13.7 h; *p* = 0.009; effect size, 0.91; power (1 − *β*), 0.76; ESBL-nonproducing *E. coli* FMOX or CMZ group: 28.5 ± 18.5 h; *p* = 0.014; effect size, 0.78; power (1 – *β*), 0.71]. There were no significant differences between the ESBL-nonproducing *E. coli* CTX and ESBL-nonproducing *E. coli* FMOX or CMZ groups. After the initiation of FMOX or CMZ treatment, the time to alleviation of fever significantly decreased to 25.9 ± 17.3 h [*p* = 0.006; effect size, 0.9; power (1 – *β*), 0.92] and 26.1 ± 8.84 h, respectively, [*p* = 0.012; effect size, 0.71; power (1 – *β*), 0.35] in the ESBL-producing *E. coli* CTX group.

## Discussion

**Fig. 4:**
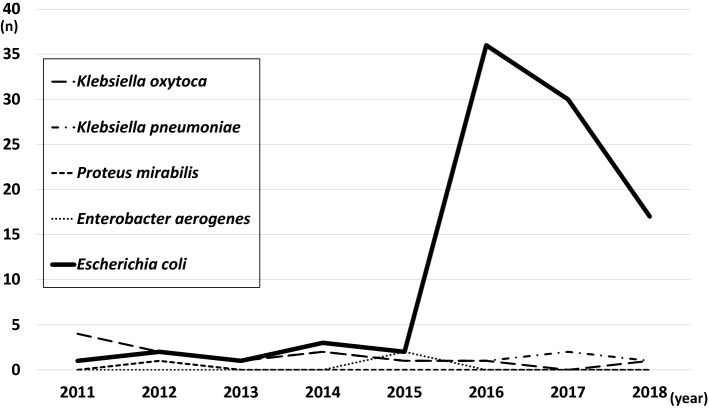
Incidence of ESBL-producing bacteria in urine sample. The number of cases in which ESBL-producing bacteria were detected in urine samples at our hospital between 2011 and 2018 are shown. *ESBL* extended-spectrum beta-lactamase

During this study period, the total number of patients hospitalized in our department decreased; however, the number of UUTI patients dramatically increased from that in 2016. The demographics of UUTI patients indicated that most cases of UUTI occurred within 6 months of life; the patients in our study were age-matched and sex-matched with Japanese children in previous reports [10]. The major cause of UUTI was *E. coli*, as previously reported; however, ESBL-producing *E. coli* caused 60.5% of all UUTI cases. Such a large prevalence of community-acquired ESBL-producing *E. coli* has not been reported previously in Japan [3, 10]. During this period, other ESBL-producing bacteria species such as *Klebsiella pneumoniae* or *Klebsiella oxytoca* were detected in up to five cases per year and were not increased at our hospital (Fig. 4).

Previous reports showed that recent use of antibiotics, previous hospitalization, presence of VUR, long-term use of prophylaxis, and history of UTI were risk factors for UTI caused by ESBL-producing *E. coli*. [5] However, in this study, we surveyed the first incidence of UUTI and found no significant differences in sex or age at the time of hospitalization between the ESBL-nonproducing *E. coli* group and ESBL-producing *E. coli* group. UUTI caused by ESBL-producing *E. coli* were increased in patients without changes in their background characteristics. Therefore, it is likely that some pathogenic factor of the bacteria changed.

In this study, the O antigens of ESBL-producing *E. coli* from stool samples were O1, O6, and O25, which are thought to have uropathogenicity, and the rate of O6 antigens dramatically increased in 2015. A previous report showed that the colonization of ESBL-producing organisms in the rectum has been associated with a high risk for the development of an infection due to ESBL-producing bacteria [11]. Moreover, the O6 antigen was important for the colonization of the urinary tract and survival in serum [12]. Additionally, the antibiotic susceptibilities of ESBL-producing *E. coli* with the O6 antigen were consistent with those of ESBL-producing *E. coli* detected in the urine samples of children. These data showed that ESBL-producing *E. coli* with the O6 antigen colonized in the intestine and caused UUTI in children.

A genomic analysis of ESBL typing, virulence genes, and PFGE was performed for nine samples of ESBL-producing *E. coli* from pediatric UUTI patients. Eight of nine ESBL-producing *E. coli* analyzed exhibited the same O6 antigens, antibiotic susceptibility, ESBL genotype (CTX-M-27), and virulence genes, including that for type 1 fimbriae. The ESBL genotype CTX-M-27 is a subgroup of CTX-M-9. The CTX-M-9 group comprises the most common types of ESBLs [3]. A previous report also showed that the CTX-M-27 subgroup has distinctive antibiotic resistance profiles and virulence profiles [4].

Virulence genes have been detected and are associated with adhesion systems, toxins, and siderophores. These virulence factors facilitate host invasion and interfere with the host defense system. Type 1 fimbriae (*fimH*) specifically has an adhesion system that facilitates attachment to urinary tract epithelial cells and helps induce inflammation [7]. These results suggested that *E. coli* with the O6 antigen and several urinary tract pathogenesis factors acquired the bla_CTX-M-27_ multi-drug resistance gene. Additionally, ESBL-producing strains were found to be similar during the PFGE analysis. Previous reports of ESBL-producing *Klebsiella pneumoniae* outbreaks have also shown dissemination of a single clone of a genotypically identical organism [1].

Previously, the rates of ESBL-producing bacilli have been low in Japan [1, 3]. However, these rates have increased in neighboring countries [1, 2], and ESBL-producing bacteria have been detected not only at medical centers but also in food, animals, and travellers [2, 13]. Previous studies performed in Germany showed that ESBL-producing bacilli in the stool dramatically increased after traveling, especially in travellers returning from Asian countries [13].

Our hospital is located in an area of Japan that has a dramatically increasing number of residents who are foreign-born individuals from South American and Asian countries. Until 2014, the total population of our city was decreasing. However, the population of foreign-born individuals doubled within 3 years, and it has continued to increase more than 10% per each year. Therefore, the ratio of the population is dramatically changing [14]. It is possible that the movement of people, animals, and materials has led to the increase in ESBL-producing *E. coli* to the same degree as that in other countries [1, 2, 13]. Such ESBL-producing bacilli outbreaks can happen anywhere. However, the reason for such an outbreak of UUTI caused by ESBL-producing *E. coli* is unclear; therefore, further studies should be conducted.

Generally, carbapenems were the most consistently used treatment against ESBL-producing bacteria. However, their overuse is concerning because it can lead to their resistance [1, 2]. Some reports showed that aminoglycoside agents [15], clavulanate/amoxicillin [16], and cephamycins such as FMOX and CMZ are effective against ESBL-producing bacteria [17, 18].

In this study, data regarding the time of hospitalization showed no differences in WBC, CRP, PCT, or relapse frequency. However, the results showed that the time until alleviation of fever from the beginning of treatment was significantly different between the ESBL-producing *E. coli* CTX group and the ESBL-nonproducing *E. coli* CTX group. Therefore, we used FMOX or CMZ for treating patients who were hospitalized with a suspected UUTI and those with Gram-negative bacilli found in the urinary Gram stain. The results showed that the use of usual doses of FMOX and CMZ significantly shortened the time to alleviation of fever without complications.

A previous study reported that UUTI caused by ESBL-producing *E. coli* despite a longer fever course after inappropriate antibiotic treatment did not increase cases of renal scarring. This might have been achieved by the pharmacodynamics/pharmacokinetics of the urine concentration. Therefore, changing empiric therapy to another treatment is not necessary [19]. However, other studies showed that the persistence of fever and delayed treatment effectiveness led to the risk of renal dysfunction [20, 21] and long hospitalizations, which increased medical expenses. Moreover, inappropriate empiric therapy focuses little attention on bacteremia associated with UUTI. It is sometimes difficult to find bacteremia associated with UUTI early enough in pediatric patients. Furthermore, recent reports showed increased cases of bacteremia associated with UTI caused by ESBL-producing bacilli, which is a risk factor for death [22, 23]. In this study, more ESBL-producing *E. coli* acquired plural pathogenesis factors and cases of bacteremia occurred.

Some reports showed that cephamycins such as FMOX and CMZ are effective as empirical and definitive therapy for patients with ESBL-producing *E. coli* bacteremia [17, 18]. FMOX and CMZ were also useful as the initial treatment for UUTI when ESBL-producing *E. coli* was suspected. However, even with different strains, the use of FMOX led to resistance to ertapenem, which is one of the carbapenems, after long-term administration due to ESBL-producing bacilli [24]. Therefore, it is necessary to focus on antibiotic susceptibility to ESBL-producing bacteria at each hospital.

## Limitations

Our study had many limitations. We conducted a retrospective review of our patients’ medical records; therefore, maternal and neonatal medical records of those who were born at other hospitals were not available for review. Medications prescribed by other hospitals and other factors before the first UUTI were not assessed. Therefore, exposure to antibiotics before the first UUTI could have altered the results of our study. Furthermore, data regarding the beginning of the UUTI before hospitalization were lacking. Therefore, further prospective studies are necessary to explore ESBL-producing *E. coli* and UUTI in children.

## Conclusion

Our study suggested that UPEC, which includes the O6 antigen and virulence genes, acquired the *bla*_CTX-M-27_ multi-drug resistance gene and caused UUTI in children.

ESBL-producing bacterial infections are community-acquired and are spreading worldwide. Therefore, ESBL-producing *E. coli* should be considered even for the first contraction of an infectious disease in children. However, carbapenems should be used carefully for the treatment of infectious diseases caused by ESBL-producing bacilli. Therefore, it is important to focus on antibiotic susceptibility when selecting appropriate antibiotics to prevent antibiotic drug resistance.
